# Autism screening and diagnosis in children with congenital heart disease

**DOI:** 10.3389/fped.2026.1839114

**Published:** 2026-06-16

**Authors:** Nuria Lisset Ontiveros Perez, Pamela M. Rios, Virginia A. Marchman, Ramkumar Aishworiya, Stella Firth Wang, Heidi M. Feldman

**Affiliations:** 1 Department of Pediatrics, Stanford University School of Medicine, Palo Alto, CA, United States; 2 Department of Psychology, Stanford University, Stanford, CA, United States; 3 Child Development Unit (CDU), Khoo Teck Puat-National University Children’s Medical Institute, National University Hospital, Singapore; 4 University of Washington, Seattle, WA, United States; 5 Cardiovascular Institute, Stanford University, Stanford CA, United States

**Keywords:** autism, autism risk, congenital heart disease, M-CHAT-R/F, public health

## Abstract

**Aim:**

This study determined the prevalence of positive autism screening results at 18-30 months of age and the presence of an autism diagnosis among children with congenital heart disease (CHD).

**Methods:**

Secondary analysis of data from Stanford site of California Perinatal Quality Care Collaborative and Lucile Packard Children's Hospital electronic health records. Participants (*N* = 94) were children born between 2016-2020 with CHD who required surgery before discharge from the intensive care unit and had a High-Risk Infant Follow-Up (HRIF) visit at age 18-30 months. Heart disease was classified as cyanotic or acyanotic. Outcomes were results of the Modified Checklist for Autism-Revised/Follow-up (M-CHAT-R/F) at the HRIF visit and evidence of a subsequent autism diagnostic code. We compared sociodemographic, clinical, and medical factors across screen results and autism diagnosis.

**Results:**

Prevalence of positive autism screens was 14.6% and autism diagnosis was 11.7%. The factor associated with screen results was age; children with positive screens were younger than children with negative screens. Use of public insurance and Risk Stratification for Congenital Heart Surgery-2 scores were higher in children with autism. Performance of M-CHAT-R/F in relation to autism diagnosis showed sensitivity of 66%, positive predictive value of 57%, and higher specificity and negative predictive values.

**Conclusion:**

In this sample, children with CHD were >3 times more likely to have positive autism screens and the diagnosis of autism than children in the general population. Early screening for autism is critical in children with CHD to promote early diagnosis and intervention.

## Introduction

Congenital heart disease (CHD) is the most common congenital anomaly in children, affecting 1-2% of live births in the U.S (1). About 25% of children with CHD require surgical repair within the first year of life (1). CHD's are typically classified into cyanotic and acyanotic depending on systemic levels of blood oxygen saturation. Approximately 20-25% of patients with CHD's have cyanotic lesions; these children typically require major surgeries in the early years of life (2). Previous literature shows that children with CHD are more susceptible to neurodevelopmental disorders than children in the general population (1).

Autism is a neurodevelopmental condition that affects an individual's social, behavioral, and developmental health (3). Current estimates show that autism affects about 3% of the general U.S. population (4). Studies have shown a higher prevalence of autism in children with CHD than in the general population, with rates of autism ranging from 3.2% to 5.9% (1); children with CHD over age 3 years are at elevated risk for the diagnosis of autism compared to children in the general population (5). However, extant literature on autism among children with CHD remains limited, with a paucity of literature regarding autism risk in the toddler period and among the population of children with severe CHD requiring early surgical intervention. Children with cyanotic CHD and/or requiring major surgical repair early in life may be at increased likelihood of autism given the severity of their hemodynamic status and the known risk of developmental consequences of major surgeries in the early childhood period due to multiple factors, including general anaesthesia (6, 7).

In California, the High Risk Infant Follow-Up (HRIF) programs enroll children with CHD who require cardiac surgery before their discharge from the intensive care unit for follow-up after hospital discharge to monitor developmental health. Assessment protocols include autism screening when children reach appropriate ages. All data forms and comprehensive assessments performed at HRIF clinic visits are completed by the evaluating clinicians and sent to the California Perinatal Quality Care Consortium (CPQCC), creating thorough records for each child that include sociodemographic, clinical and medical factors and results of autism screenings. At the Stanford site of the CPQCC, the Modified Checklist for Autism in Toddlers-Revised with Follow-Up (M-CHAT-R/F) is typically administered between 18-30 months of age with the recommended interview as appropriate at these HRIF visits (8). The actions based on the results of screening are at the discretion of the clinician and family in a process of shared decision-making. Most who are at a higher likelihood for autism are referred for further evaluations, typically outside of the HRIF clinical program and occasionally outside of the Stanford Medicine Children's Health Network. However, the clinical reasons for not referring children for evaluation are not reliably found in the medical records.

We analyzed the CPQCC records of children with CHD that attended the Stanford site HRIF clinic and reviewed their medical records to obtain information not included in the CPQCC records: the CHD subgroup type, validation of autism screening results, and presence of autism diagnosis at ages older than the HRIF visit. We asked the following research questions:

1. In this sample of children with CHD who require surgery before discharge from the neonatal hospitalization, what is the likelihood of a positive autism screen at 18-30 months?
  - What sociodemographic and clinical factors contribute to this likelihood?
  - Does the likelihood of a positive screen differ in children with cyanotic or acyanotic heart disease?
2. What is the prevalence of a diagnosis of autism in this sample?
  - What sociodemographic and clinical factors are associated with the diagnosis of autism?
  - Does the likelihood of a diagnosis of autism differ in children with cyanotic or acyanotic heart disease?
  - What are the performance metrics of the M-CHAT-R/F in prediction of the diagnosis of autism in this sample of children with CHD?

We hypothesized that the children with CHD would have an elevated likelihood of screening positive for autism risk and a higher prevalence of autism diagnosis than the general population. If correct, these results reinforce the importance of autism screening and prompt diagnostic evaluations, if necessary. This analysis also evaluates whether the M-CHAT-R/F is an appropriate screening instrument in this population, since the measure was not validated on this specific clinical group. The ultimate goal of these efforts is to improve neurodevelopmental outcomes in this medically-vulnerable group.

## Methods

### Design and participants

Secondary analysis of data collected at Stanford Medicine Children's Health HRIF clinic of the CPQCC. Inclusion criteria were infants: (1) born ≤ 42 weeks gestation (GA, range = 23–42) between 2016–2020, (2) with congenital heart disease requiring surgery before discharge home from the neonatal hospitalization, and (3) who completed the 18-30 month HRIF visit at Stanford Medicine Children's Health. Exclusion criteria were the following: chromosomal or genetic conditions, such as Down syndrome; other major congenital anomalies, such as congenital diaphragmatic hernia; blindness or deafness. We only included children with CHD without any contributing or co-occurring genetic conditions and other organ congenital anomalies to minimise the possibility that autism was due to the underlying genetic abnormality, instead of CHD and its management.

### Procedure

At the Stanford HRIF clinic, the M-CHAT-R/F is typically administered between 18-30 months of age, with the recommended interview conducted if needed, for respondents with initial scores between 3-7 (8). We classified the participants in two groups based on scores on the M-CHAT-R/F: (1) positive-screens, indicating an elevated likelihood of autism, defined as scores ≥ 2 after interview or > 7 pre-interview and (2) negative-screens, indicating low likelihood of autism defined as initial scores ≤ 2 or post-interview scores < 2. We extracted the following sociodemographic data from each child's record: sex, child age at M-CHAT-R/F assessment, mother's age, race, ethnicity, and insurance status (public versus private). We extracted the following general clinical factors from the CPQCC data set: gestational age at birth, birth weight, length of neonatal hospital stay, and timing of early heart surgery.

We then conducted a chart review of all electronic medical records from Lucile Packard Children's Hospital of children with CHD, whether or not the children were screened for autism between 18 and 30 months of age. In this chart review, we extracted the specific CHD diagnosis and the type and timing of the first surgery before hospital discharge. We looked for evidence of a referral of diagnostic evaluation and use of early intervention services of any type among the children with positive screen results. We classified each CHD diagnosis into cyanotic (reduced blood oxygen saturation levels) and acyanotic (appropriate blood oxygen saturation levels). We classified the surgery as “neonatal” if the first surgery occurred within the first month of life and “post-neonatal” if the surgery occurred when the child was older than one month. We scored the type of initial surgery according to the Risk Stratification for Congenital Heart Surgery (RACHS-2) tool for ICD-10 coded data (RACH-2) (9). For all children, including both those who were screened and those who were not screened, we determined if they ever received the diagnosis of autism, based on the use of the appropriate ICD 10 code for autism spectrum disorder or ASD (F84.0), by any of the child's clinicians and the age at time of first mention of this diagnosis.

### Data analysis

As a preliminary analysis, we compared the subgroup that was screened to the subgroup that was not screened on child sex, gestational age at birth, race, ethnicity and insurance status using independent sample t-tests and chi-square tests. Using all children who received the M-CHAT-R/F screening as part of their HRIF visit, we next compared the screen-positive and screen-negative groups on demographic factors, clinical factors, and CHD group (cyanotic or acyanotic) using independent sample t-tests, chi-square tests, Wilcoxon rank sum tests, and Fisher's test as appropriate.

For the analysis of autism diagnosis, we used the entire sample, including both the children who did and did not receive autism screening. We compared the children who did and did not have evidence of the diagnosis of autism on the same demographic factors, clinical factors, and CHD group (cyanotic or acyanotic) using independent sample t-tests, chi-square tests, Wilcoxon rank sum tests, and Fisher's test as appropriate.

We derived sensitivity, specificity, positive and negative predictive values of the M-CHAT-R/F in predicting diagnosis of autism within the group of children who were screened. We created a Receiver Operating Curve (ROC) analysis to assess the area under the curve (AUC) as a summary of the performance of the screening measure. We generated the confidence interval of the AUC using the Hanley-McNeil Method (10). All analyses were run in R (Version 4.4.1).

## Results

**Figure 1:**
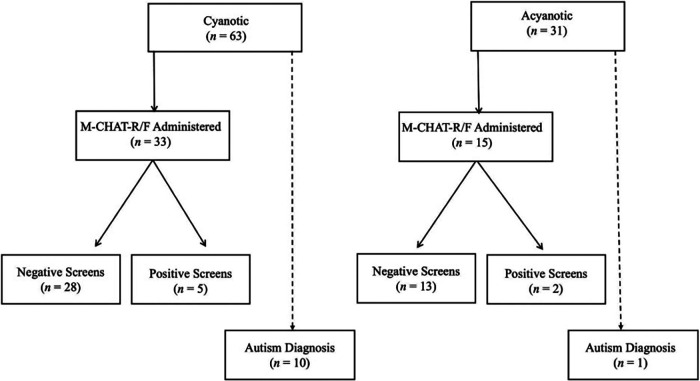
Participant Flow Diagram Representing two CHD subgroups, Cyanotic and Acyanotic with the M-CHAT-R/F screening results and Autism Diagnosis.

A total of 129 children were registered in the CPQCC data set as having CHD and a HRIF clinic visit at 18-30 months of age. Of these, 35 were excluded from the analyses on the basis of (1) having a chromosomal or genetic conditions (*n* = 14, of which 9 had Down syndrome) or no evidence of cardiac surgery (*n* = 21, such as patent ductus arteriosus treated medically, CHD without surgery, or CHD with surgery unrelated to CHD) (Figure 1). Of the 94 remaining children, 48 had the M-CHAT-R/F administered and 46 did not. There were no statistically significant differences on sociodemographic or clinical variables between those screened versus those not-screened.

Of those who received the screening test, 41 (85.4%) resulted in negative-screens and 7 (14.6%) in positive-screens. Table 1 presents descriptive and comparative data for sociodemographic and clinical factors as a function of M-CHAT-R/F screening groups. There were no statistically significant differences between the positive- and negative-screen group on child sex, mother's age, race, ethnicity, or use of public insurance as a proxy for socioeconomic status (all p ns). However, the children with positive screen results were younger than the children with negative screen results (*p* < .001). There were also no statistically significant differences between the positive- and negative-screen group on clinical variables, including length of hospital stay, gestational age at birth, birthweight, neonatal versus post-neonatal operation, or RACHS-2 score.

**Table 1:** Demographic and clinical factors associated with M-CHAT-R/F screening results (*N* = 48).

Variables	M-CHAT-R/F Negative *n*(%) or M(SD) *N* = 41	M-CHAT-R/F Positive *n*(%) or M(SD) *N* = 7	*t*, OR (CI), W	*p*
Demographic variables				
Child Sex (Male)	25 (61%)	5 (71%)	0.63 (.05−4.45)	0.70
Child's Age at Assessment (Months)	23.5 (2.7)	19.9 (1.2)	5.92	**<0.001**
Mother's Age (Years)	30.5 (5.5)	29.1 (6.6)	0.50	0.63
Race (Non-white)	29 (71%)	4 (57%)	1.79 (.23−12.43)	0.66
Ethnicity (Hispanic)	19 (46%)	3 (43%)	1.15 (.17−8.85)	1.00
Insurance (Public)	20 (49%)	3 (43%)	0.79 (.10−5.33)	1.00
Clinical Variables				
Length of stay (Days)	57.2 (61.2)	88.7 (78.5)	−1.01	0.34
Gestational Age at birth (Weeks)	35.9 (5.5)	35.9 (5.2)	−0.00	1.00
Birth Weight (Grams)	2736.1 (1086.1)	2624.0 (1145.5)	0.24	0.82
Surgery (% Neonatal, before age 1 month)	28 (68%)	5 (71%)	0.86 (.07−6.20)	1.00
RACHS-2* score	2.7 (1.3)	3.1 (1.5)	117.0	0.43

* Risk stratification for congenital heart surgery.

Bold values indicate statistical significance with p < .05.

Figure 1 includes the participant flow diagram as a function of CHD group. Of 63 in the cyanotic CHD group, 33 (52.4%) had the M-CHAT-R/F completed, 28 (84.8%) had a negative-screen and 5 (15.2%) had a positive-screen. Of 31 in the acyanotic CHD group, 15 (48.4%) had the M-CHAT-R/F completed, 13 (86.7%) had a negative-screen, and 2 (13.3%) had a positive-screen. All children with positive screens were receiving early intervention services.

Among the entire sample (*N* = 94), chart review revealed that 11 children had a confirmed autism diagnosis (11.7% prevalence rate). Children were aged 2 to 5 years at the first mention of the diagnostic code in the child's medical record. Figure 1 also shows that within the cyanotic subgroup, 10 (15.9%) had an autism diagnosis and within the acyanotic subgroup 1 (3.2%) had an autism diagnosis. This difference in prevalence rate did not reach the level of statistical significance. Table 2 compares the children who were diagnosed with autism (autism positive) and those who were not (autism negative). Among the sociodemographic variables, the only one with a statistical difference was use of public insurance (*p* = .003). Among the clinical variables, children with autism had a higher RACHS-2 score than those who did not have autism (*p* = .02).

**Table 2:** Demographic and clinical factors associated with presence of autism ICD-10 diagnostic code in the electronic health record (*N* = 94).

Variables	Autism Negative *n*(%) or M(SD) *N* = 83	Autism Positive *n*(%) or M(SD) *N* = 11	*t*, OR (CI), W	*p*
Demographic variables				
Child Sex (Male)	49 (59%)	9 (82%)	0.32 (.03–1.71)	0.20
Mother's Age (Years)	31.3 (5.6)	30.1 (4.8)	0.75	0.47
Race (Non-white)	60 (72%)	7 (64%)	1.48 (.29–6.51)	0.72
Ethnicity (Hispanic)	46 (55%)	4 (36%)	0.46 (.09–2.00)	0.34
Insurance (Public)	35 (42%)	10 (91%)	13.40 (1.76–605.40)	**0.003**
Clinical Variables				
Length of stay (Days)	55.2 (61.0)	71.6 (59.7)	−0.86	0.41
Gestational Age at birth (Weeks)	36.3 (4.9)	37.0 (2.2)	−0.81	0.42
Birth Weight (Grams)	2777.2 (1052.2)	2945.3 (676.9)	−0.72	0.48
Surgery (% Neonatal, before age 1 month)	53 (64%)	10 (91%)	0.18 (.00–1.37)	0.09
RACHS-2* score	2.6 (1.2)	3.6 (1.4)	263.5	**0.02**

* Risk stratification for congenital heart surgery.

Bold values indicate statistical significance with p < .05.

**Figure 2:**
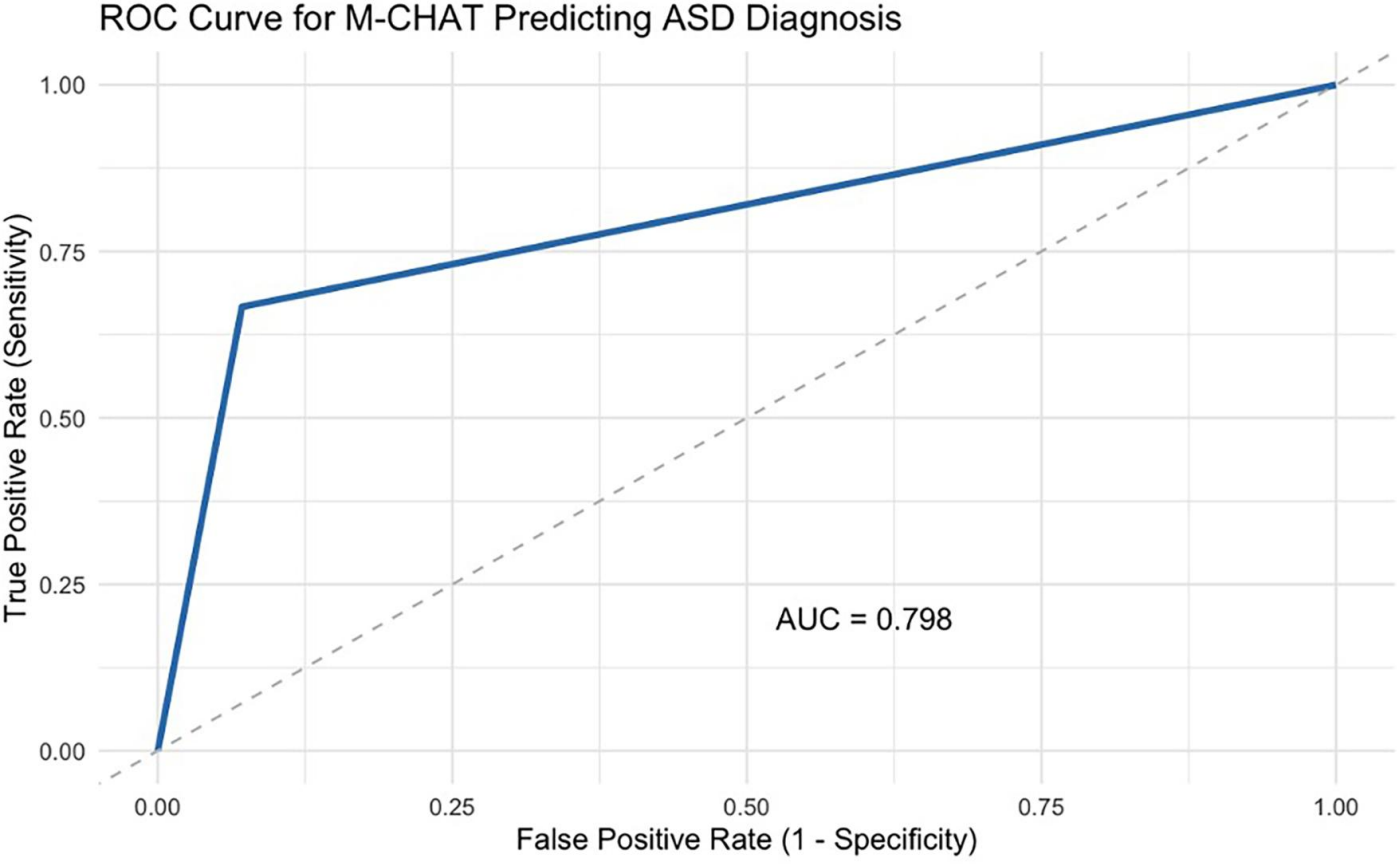
Receiver Operator Characteristics (ROC) curve analysis showing overall model fit as Area Under the Curve (AUC) and sensitivity and specificity for the M-CHAT-R/F Predicting Autism Diagnosis in this sample of children with Congenital Heart Disease requiring surgery.

To assess if the M-CHAT-R/F is an appropriate tool to use for autism screening in a sample of children with CHD requiring surgery before hospital discharge, we calculated psychometric properties and an ROC comparing results of screening and actual autism diagnosis for the subsample who had M-CHAT-R/F screening results (*n* = 48). The M-CHAT-R/F demonstrated a sensitivity of 67% CI [0.33–1.00], correctly identifying 4 of 6 children with ASD, and a specificity of 93%, CI [0.83–1.00] correctly identifying 39 of 42 children without ASD. Among children with a positive M-CHAT-R/F result, 4 of 7 children were diagnosed with ASD, with a positive predictive value of 57%, CI [0.18–0.90]. Among children with a negative M-CHAT-R/F result 39 of 41 children did not receive an ASD diagnosis with a negative predictive value of 95%, CI [0.83–0.99). Narrow confidence intervals for specificity confirm that we had adequate with this sample size; broad confidence intervals for sensitivity are consistent with the lack of power to reliably confirm sensitivity with this sample size (11). As shown in Figure 2, the AUC was 0.80, CI [0.64-0.96], indicating good accuracy.

## Discussion

We sought to determine the prevalence of positive M-CHAT-R/F screening results and subsequent diagnoses of autism in a sample of children with relatively severe CHD, as evidenced by the need for surgery during the initial neonatal hospitalization. The percentage with positive screens approached 14.6%, with similar rates of positive screens in children with cyanotic and acyanotic CHD. This rate is almost three times higher than the 4.9% positive screen rate reported in the general population (8). It is also modestly higher than the 12.2% (12) reported rate in children born preterm, another group with a prolonged neonatal hospital stay, multiple medical procedures, and disrupted early parent-child relationships. The percentage with a diagnosis of autism was 11.7%. While the percentage of a positive diagnosis was higher among those in the cyanotic CHD group (15.9%) than those in the acyanotic CHD group (3.2%), the difference did not reach statistical significance, likely related to the sample size. This rate is more than three times the current reported prevalence of autism in the general population of 3.2% (Shaw et al., 2025). It is also higher than the reported rate of autism in children born extremely prematurely of 6.1% (13). These findings reinforce the importance of prompt autism screening and diagnostic evaluation for autism when appropriate in children with CHD, especially those with severe conditions requiring surgical intervention before discharge from an initial neonatal hospitalization.

Child age at screening was associated with screen results; those with positive screens were younger than those with negative screens. This result may be related to the better accuracy of the M-CHAT-R/F at 24 to 30 months than at 18 months (14). However, the result may also relate to clinical practices in the HRIF program. When clinicians are concerned about a child's developmental standing, they often schedule the child earlier in the target window for evaluation. In that way, the clinicians can assure that the child receives all evaluations and is referred to therapeutic services at as young an age as possible.

Child insurance status was associated with diagnosis results; those with evidence of autism were highly likely to be receiving public insurance. This result likely relates to California health insurance policies that make children with disabilities, such as autism, eligible for public insurance through mechanisms other than household income, including institutional deeming and participation in California Children's Services. In children with disabilities, public insurance is a less accurate indicator of socioeconomic status than it is in children without disabilities.

RACHS-2 scores were also associated with diagnostic results; children with autism had higher RACHS-2 scores than children without autism. RACHS-2 scores are a validated risk stratification tool that was developed to predict in-hospital mortality for pediatric cardiac surgeries (9). This novel finding suggests that factors associated with in-hospital mortality are also important in long-term neurodevelopmental outcomes.

The performance of the M-CHAT-R/F in relation to an eventual autism diagnosis showed stronger specificity (92%) and negative predictive value (95%) than sensitivity (66%) and positive predictive value (57%). The AUC of 0.80 indicates that the screening test has acceptable overall accuracy at distinguishing between those with and without autism. Given the modest sample size of this study, the confidence interval indicates that the screening test could range from fair to excellent accuracy.

Studies reporting on the psychometric properties of the M-CHAT-R/F in the general population typically find sensitivity and negative predictive value are lower than specificity and positive predictive value (8). However, the psychometric values vary as a function of the particular study. Real-world samples, which assess non-experimental clinical populations, have found even lower sensitivity than we found in this sample (15, 16). In addition, the results here on the performance of the M-CHAT-R/F are comparable to those in a recent meta-analysis (17). Overall, we conclude that the M-CHAT-R/F is an acceptable tool for screening in this population, though the rate of false positives may be elevated and further research is warranted.

Limitations of the current analysis are (1) potential bias by restricting the sample to children who attended the HRIF visit at 18-30 months, (2) the modest sample size and (3) the relatively low proportion of participants who had an autism screening completed at 18-30 months at an HRIF visit due to suspension of autism screening during the COVID-19 pandemic, when paper forms were not filled out by parents whose children were evaluated via telehealth. Another limitation is that the confirmed autism diagnosis was extracted from medical record review. The diagnosis could have been missed if not appropriately coded within the visit diagnoses or problem list or if the diagnostic evaluation was completed elsewhere and the child did not return for a subsequent visit to our hospital. In either case, these factors would have likely led to an underestimate of autism prevalence rates, particularly in the acyanotic CHD subgroup. Finally, we had no information on the developmental levels of the children with and without autism, though we confirmed that the children with positive autism screens were all enrolled in early intervention services, suggesting that developmental delays meeting eligibility criteria had been identified.

## Conclusion

This study of children with CHD requiring surgery before discharge from the neonatal hospitalization found that the prevalence of autism-likelihood based on screening results and the prevalence of the diagnosis of autism was more than 3 times those rates within the general population. The M-CHAT-R/F had acceptable accuracy with strong specificity and negative predictive value, though modest sensitivity and positive predictive value. These results support reliable and routine autism screening for children with CHD at 18-30 months of age and prompt diagnostic evaluations for those with positive screen results. Future research should explore the properties of other screening tests in this population and possible clinical variables associated with positive screening and diagnostic results and determine if changes in clinical practice might reduce autism prevalence in the future.

## Data Availability

The raw data supporting the conclusions of this article will be made available by the authors, without undue reservation.
